# Block-Based Compression and Corresponding Hardware Circuits for Sparse Activations

**DOI:** 10.3390/s21227468

**Published:** 2021-11-10

**Authors:** Yui-Kai Weng, Shih-Hsu Huang, Hsu-Yu Kao

**Affiliations:** Department of Electronic Engineering, Chung Yuan Christian University, Taoyuan 32023, Taiwan; genoside0079@gmail.com (Y.-K.W.); darren.kao.s@cycu.org.tw (H.-Y.K.)

**Keywords:** compression formats, convolutional neural networks, data volume, digital circuits, edge computing, logic design

## Abstract

In a CNN (convolutional neural network) accelerator, to reduce memory traffic and power consumption, there is a need to exploit the sparsity of activation values. Therefore, some research efforts have been paid to skip ineffectual computations (i.e., multiplications by zero). Different from previous works, in this paper, we point out the similarity of activation values: (1) in the same layer of a CNN model, most feature maps are either highly dense or highly sparse; (2) in the same layer of a CNN model, feature maps in different channels are often similar. Based on the two observations, we propose a block-based compression approach, which utilizes both the sparsity and the similarity of activation values to further reduce the data volume. Moreover, we also design an encoder, a decoder and an indexing module to support the proposed approach. The encoder is used to translate output activations into the proposed block-based compression format, while both the decoder and the indexing module are used to align nonzero values for effectual computations. Compared with previous works, benchmark data consistently show that the proposed approach can greatly reduce both memory traffic and power consumption.

## 1. Introduction

Nowadays, convolutional neural networks (CNNs) [1,2] have been widely used in many application fields, such as computer vision [3,4], signal processing [5,6] and image processing [7,8]. Note that a CNN is composed of multiple layers. Most of the layers in a CNN are convolutional (CONV) layers, which consume a large portion of the overall execution time. To speed up the intensive computations of the CONV layers, a lot of customized hardware accelerators [9,10,11,12,13,14,15,16,17,18] have been proposed to deal with this problem.

In addition to the intensive computations, the large data volume of a CNN model is also an important issue for the design of a hardware accelerator [19,20,21]. As discussed in [19], for a hardware accelerator, most of the energy consumption is spent on off-chip memory (i.e., DRAM). To reduce the energy consumption of a hardware accelerator, there is a demand to reduce the data movement to off-chip memory. In particular, for the edge computing, since the hardware accelerator is designed with a stringent power constraint (energy constraint), this issue becomes more important.

In many CONV layers, as a consequence of the Rectified Linear Unit (ReLU), a large fraction of the activations are zero values. Here, we use six pre-trained CNN models in Keras [22], including vgg16, ResNet50, ResNet50v2, MobileNet, MobileNetv2 and DenseNet121, for illustration. As shown in Table 1, for each CNN model, the activation sparsity (i.e., the percentage of zero value among all activations) is at least 33.1%. Note that ineffectual multiplications (i.e., multiplications by zero) can be skipped. Therefore, Cnvlutin [23] tries to exploit the activation sparsity to reduce the data volume.

In Cnvlutin [23], the sparse activations are represented by a compression format, which only records the values and the indices (i.e., the spatial information) of the nonzero activation values. As a result, the data volume (that needs to be transferred from off-chip memory) can be significantly reduced. To handle the compression format (i.e., to handle the alignment of irregularly distributed nonzero activation values), an indexing mechanism is used in Cnvlutin [23].

To increase the sparsity of weights, weight pruning [24,25] can be used to remove all weights below a certain threshold value (it is noteworthy to mention that, to minimize the loss on accuracy, a costly retraining step may be required after weight pruning [24,25]). Cambricon-X [9] proposes a compression format (with corresponding indexing mechanisms) to exploit the sparsity of weights. Compared with the GPU (with the sparse library), on average, Cambricon-X [9] can achieve 10.60× speedup and 29.43× energy reduction.

Lin and Lai [26] consider both the sparsity of activations and the sparsity of weights. In their approach [26], both activations and weights are kept in a compression format. Then, a dual indexing module is proposed to check the indices of activations and weights in parallel. By using the dual indexing module, the effectual activation/weight pairs can be identified for computations. Furthermore, in [27], a single-output dual indexing module is proposed to identify the effectual activation/weight pairs in a fine-grained manner.

Previous works [9,23,26,27] exploit the sparsity to reduce both memory traffic and power consumption. Different from these previous works, in this paper, we point out the similarity of activation values. With an analysis of CNNs, we have the following two observations of activation values.

**Figure 1:**
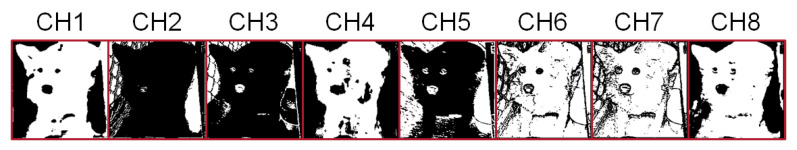
Feature maps in layer 2 of CNN model vgg16.

**Figure 2:**
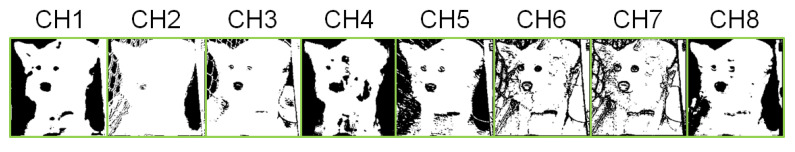
Colors of CH2, CH3 and CH5 are reversed.

- In the same layer of a CNN model, most feature maps are either highly dense or highly sparse. Take the feature maps in layer 2 of the CNN model vgg16 for illustration. Figure 1 gives the feature maps of the first eight channels in layer 2 of the CNN model vgg16. In Figure 1, a zero value is displayed in a white color, while nonzero values are displayed in a black color. Then, we can find: channels CH2, CH3 and CH5 are highly dense, while channels CH1, CH4, CH6, CH7 and CH8 are highly sparse. In other words, for the same feature map, two adjacent pixel locations are often in the same color. Thus, there is a high possibility that two adjacent pixel locations, called a block, can share the same indication bit.
- In the same layer of a CNN model, feature maps in different channels are often similar. Take the first eight feature maps in layer 2 of the CNN model vgg16 for example. As displayed in Figure 1, channels CH2, CH3 and CH5 are white dog pictures, while channels CH1, CH4, CH6, CH7 and CH8 are black dog pictures. In other words, these eight feature maps are essentially dog pictures. In particular, if the colors of CH2, CH3 and CH5 are reversed, we can obtain the eight feature maps, as shown in Figure 2. Note that these eight feature maps (displayed in Figure 2) are similar. Owing to the similarity of feature maps, we can try to consider multiple channels at the same time for compression.

Based on these two observations, we are motivated to utilize the similarity of activation values to further reduce the data volume. In other words, in addition to utilizing the sparsity, we also try to utilize the similarity of activation values to further reduce the data volume.

In this paper, to exploit both the sparsity and the similarity of activation values, we develop a block-based compression approach (i.e., block-based compression format) to store activation values. Furthermore, to support the proposed block-based compression format, we also develop an encoder, a decoder and an indexing module. The encoder is used to translate output activations into the proposed block-based compression format. Both the decoder and the indexing module are used to align nonzero activation values for effectual multiplications. Compared with previous works, benchmark data consistently show that the proposed approach can greatly reduce both memory traffic and power consumption (energy consumption).

The rest of this paper is organized as follows. Section 2 gives a survey on related works. In Section 3, we present the proposed block-based compression format and its corresponding hardware designs (including an encoder, a decoder and an indexing module). Then, in Section 4, we report the experiment results. Finally, some concluding remarks are given in Section 5.

## 2. Related Works

To exploit the parallelism in CNNs, many CNN accelerators [9,10,11,12,13,14,16,17,18,23,26,27] are designed based on the single-instruction–multiple-data (SIMD) architecture. Note that the core of convolution operation is multiplication and accumulation. Therefore, in the SIMD architecture, multiply-accumulate (MAC) engines [28,29,30] are used to support convolution operations between input activations and kernel weights. No matter if a CNN is sparse or not, the compression format cannot be directly applied to the SIMD architecture; otherwise, irregularly distributed nonzero values will break the alignment of input activations and kernel weights. To handle the compression format (i.e., to handle the alignment of input activations and kernel weights), an indexing mechanism is required.

**Figure 3:**
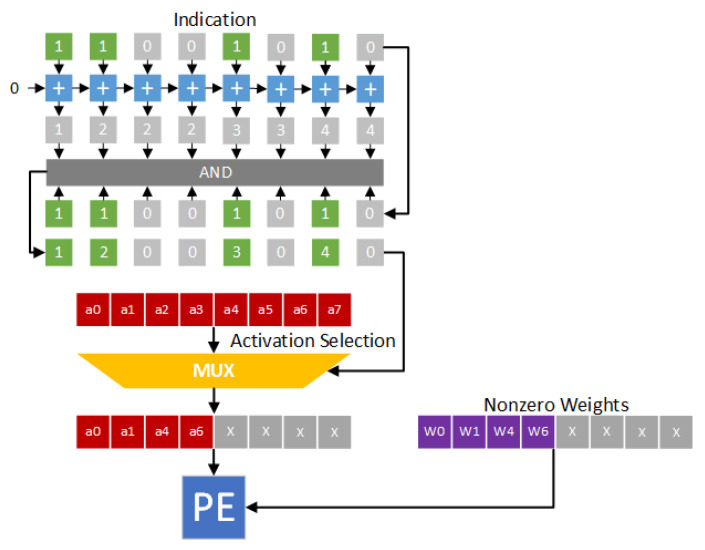
The hardware design of the direct indexing module [9].

Owing to its simplicity, direct mapping [9,26,27] is widely used as the compression format. Note that direct mapping is implemented with a bit string (called an indication string). In the indication string, each bit corresponds to an activation (or a weight) and indicates if the value is zero or not (”1” for nonzero value and ”0” for zero value). For example, in Cambricon-X [9], weights are stored in the compression format. Only nonzero weights are stored in the memory, and an indication string is used to indicate if each weight is zero or not. Figure 3 gives the hardware design of the direct indexing module. In Figure 3, weights w0, w1, w4 and w6 are nonzero values. Thus, the indication string is 11001010. We add each bit in the indication string to obtain an accumulated string. In the accumulated string, each element denotes the corresponding location. By enforcing the “AND” operation between the indication string and the accumulated string, the indexes of nonzero weights can be obtained. Therefore, as shown in Figure 3, activations a0, a1, a4 and a6 are selected. The pairs (a0,w0), (a1,w1), (a4,w4) and (a6,w6) are sent to the processing engine (PE) for performing convolution operations.

**Figure 4:**
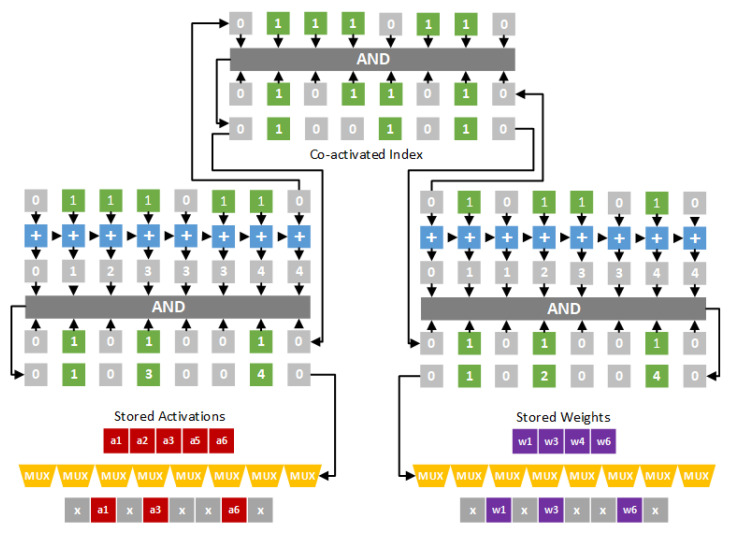
The hardware design of the dual indexing module [26].

In [26], both activations and weights are stored in the compression format. To determine effectual activation/weight pairs, a dual indexing module [26] is proposed. Figure 4 gives the hardware design of the dual indexing module. In Figure 4, activations a1, a2, a3, a5 and a6 are nonzero values, and weights w1, w3, w4 and w6 are nonzero values. Thus, the indication string of activations is 01110110, and the indication string of weights is 01011010. A bit-wise “AND” operation is applied on the two indication strings to obtain the co-activated index 01001010. Note that the co-activated index is used to mask out ineffectual activations and weghts. Therefore, as shown in Figure 4, activations a1, a3 and a6 and weights w1, w3 and w6 are selected. The pairs (a1,w1), (a3,w3) and (a6,w6) are sent to the processing engine (PE) for performing convolution operations.

**Figure 5:**
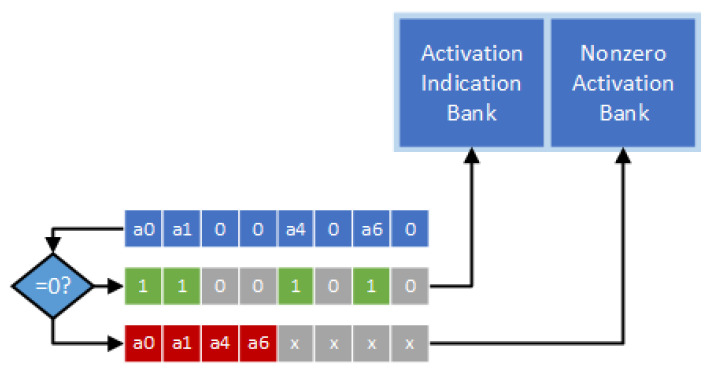
The hardware design of the encoder [26].

Note that output activations are dynamically generated during the inference process. Therefore, as described in [26], an encoder is needed to dynamically encode output activations into the direct indexing format. Figure 5 gives the hardware design of the encoder. In Figure 5, a zero-comparator is used to scan through output activations. Then, all the nonzero activation values and the indication string can be stored. It is noteworthy to mention that, although the process of encoding is sequential, it does not cause any extra cycle since it is not on the critical path [26].

## 3. Proposed Approach

In this section, we propose a block-based compression format, which utilizes both the sparsity and the similarity of activation values, to reduce the data volume. Then, we design an encoder, a decoder and an indexing module to support the proposed block-based compression format.

**Figure 6:**
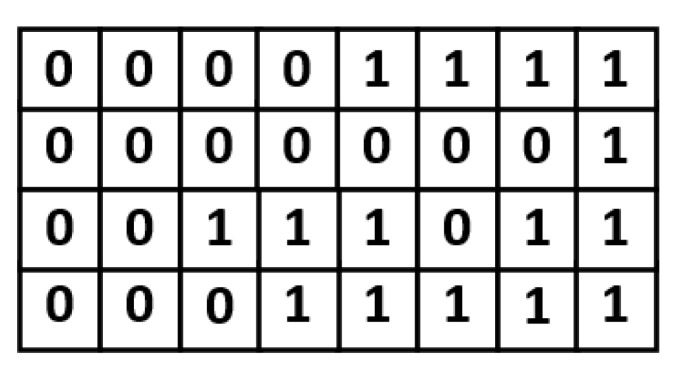
An indication matrix example.

**Figure 7:**
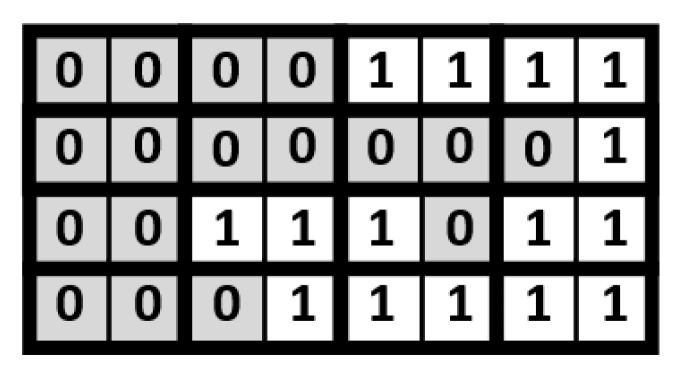
Split the indication matrix into 16 blocks.

**Figure 8:**
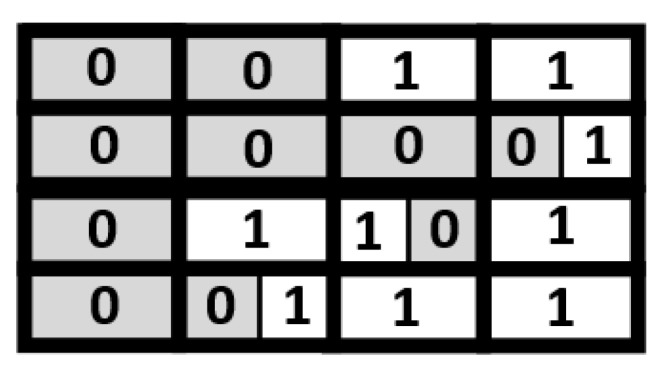
The compressed indication matrix.

In the proposed approach, an indication matrix is split into a number of 2×1 size blocks. Take the indication matrix shown in Figure 6 for illustration. This indication matrix is split into 16 blocks. As displayed in Figure 7, each block contains 2 indication bits. Note that, in each block, the values of the two indication bits are often the same. If the values of the two indication bits are the same, we can replace the two indication bits with a single indication bit. In other words, in a block, if the value of each indication bit is “1”, the two indication bits can be reduced to be a single indication bit with a binary value of “1”; if the value of each indication bit is “0”, the two indication bits can be reduced to be a single indication bit with a binary value of “0”. Figure 8 gives the corresponding compressed indication matrix.

**Figure 9:**
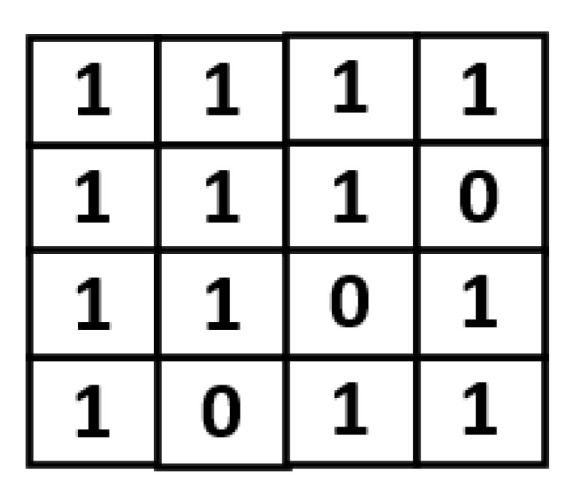
The corresponding LUT of the compressed indication matrix.

Note that the compressed indication matrix is irregular. As shown in Figure 8, some blocks have two indication bits, while others have only one indication bit. Thus, we also need a look-up table (LUT) to identify the number of indication bits of each block. Note that the LUT is a table of 1-bit marks. For each block in the compressed indication matrix, there is a corresponding mark bit in the LUT. Using the compressed indication matrix displayed in Figure 8 as an example, Figure 9 gives the corresponding LUT. For each mark bit in the LUT, the binary value “0” means two indication bits, while the binary value “1” means a single indication bit.

Note that, in the same layer of a CNN model, feature maps in different channels are often similar. Therefore, to reduce the LUT size, an LUT is shared by multiple channels. The proposed sharing method is below. For each block of a feature map (i.e., a channel), we can specify its position by a coordinate value. We say two blocks in two different feature maps (i.e., two different channels) are in the same group if and only if the two blocks have the same coordinate value. Then, for the blocks in the same group, they share the same mark in the LUT.

**Figure 10:**
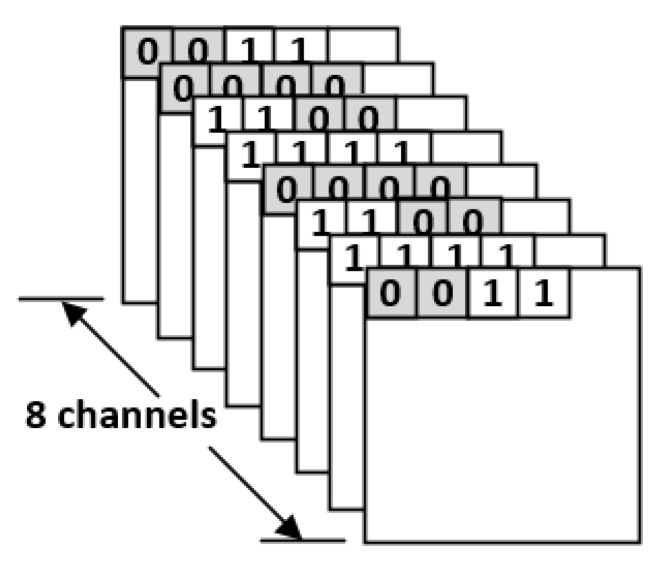
Indication matrices of eight channels.

**Figure 11:**
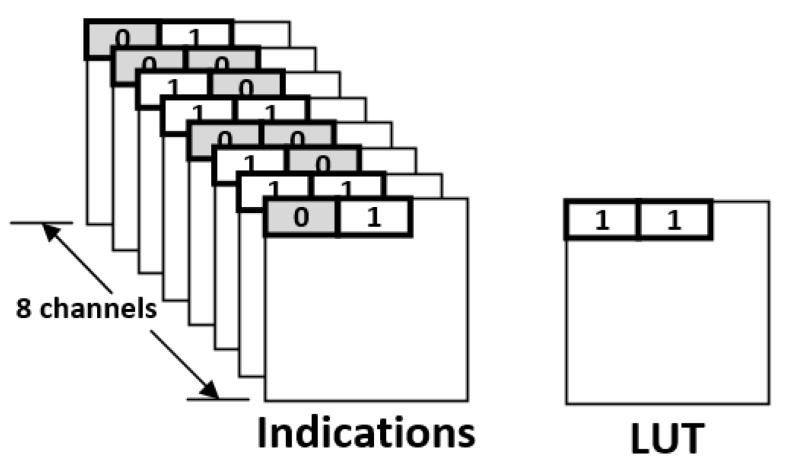
The proposed compression format.

Take the indication matrices shown in Figure 10 for example. Here we consider eight channels at the same time for compression. For each block in Figure 10, its two indication bits are the same. Thus, we can replace the two indication bits with a single indication bit. As a result, we can obtain the compression result as shown in Figure 11. Note that, in Figure 11, there are two groups: each group has eight blocks, which are from different channels and in the same coordinate value. For each group, there is a corresponding mark bit in the LUT. For each mark bit in the LUT, the binary value “0” means each block in this group uses two indication bits, while the binary value “1” means each block in this group uses a single indication bit.

It is noteworthy to mention that, since each block in the same group shares the same mark bit in the LUT, each block in the same group should use the same number of indication bits. In other words, a block in a group can use only one indication bit, if and only if all the blocks in this group can only use one indication bit. If a block in a group needs to use two indication bits, then all the blocks in this group need to use two indication bits. Fortunately, owing to the similarity of feature maps, there is a high possibility that each block in a group only needs to use one indication bit. Using CNN model vgg16 for illustration, even if we consider eight channels at the same time, there are 48.7% groups in which each block only needs to use one indication bit. In other words, for 48.7% of the groups, their corresponding mark bit value is “1”.

Algorithm 1 gives the pseudo code of the proposed block-based compression algorithm. Without loss of generality, here we assume that the number of channels is eight. In Algorithm 1, the notations $X_{1}$, $X_{2}$, $X_{3}$, $X_{4}$, $X_{5}$, $X_{6}$, $X_{7}$ and $X_{8}$ denote the eight original indication matrices, the notations $Y_{1}$, $Y_{2}$, $Y_{3}$, $Y_{4}$, $Y_{5}$, $Y_{6}$, $Y_{7}$ and $Y_{8}$ denote the eight compressed indication matrices and the notation LUT denotes the LUT. We use one-dimensional arrays to represent these indication matrices and the LUT. The notation N denotes the length of each original indication matrix, while the notation c denotes the length of each compressed indication matrix. For the sake of brevity, here we assume N is an even number. Thus, for each original indication matrix, the number of 2×1 size blocks is N/2. In other words, the length of the LUT is N/2.
**Algorithm 1:** Proposed Block-Based Compression
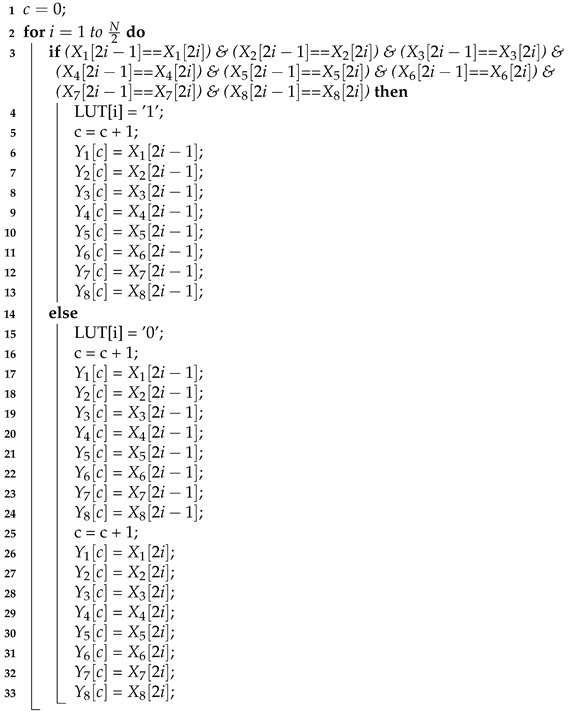


In the proposed block-based compression algorithm (as displayed in Algorithm 1), indication bit $X_{1}\left[2i-1\right]$ and indication bit $X_{1}\left[2i\right]$ are in the same 2×1 size block, where i=1,2,…, and (N/2). Here we use the pair ($X_{1}\left[2i-1\right]$, $X_{1}\left[2i\right]$) to represent this 2×1 size block. The eight blocks ($X_{1}\left[2i-1\right]$, $X_{1}\left[2i\right]$), ($X_{2}\left[2i-1\right]$, $X_{2}\left[2i\right]$), ($X_{3}\left[2i-1\right]$, $X_{3}\left[2i\right]$), ($X_{4}\left[2i-1\right]$, $X_{4}\left[2i\right]$), ($X_{5}\left[2i-1\right]$, $X_{5}\left[2i\right])$, ($X_{6}\left[2i-1\right]$, $X_{6}\left[2i\right]$), ($X_{7}\left[2i-1\right]$, $X_{7}\left[2i\right]$) and ($X_{8}\left[2i-1\right]$, $X_{8}\left[2i\right]$) belong to the same group, where i=1,2,… and (N/2). For the sake of brevity, we say the group formed by blocks ($X_{1}\left[2i-1\right]$, $X_{1}\left[2i\right]$), ($X_{2}\left[2i-1\right]$, $X_{2}\left[2i\right]$), ($X_{3}\left[2i-1\right]$, $X_{3}\left[2i\right]$), ($X_{4}\left[2i-1\right]$, $X_{4}\left[2i\right]$), ($X_{5}\left[2i-1\right]$, $X_{5}\left[2i\right]$), ($X_{6}\left[2i-1\right]$, $X_{6}\left[2i\right]$), ($X_{7}\left[2i-1\right]$, $X_{7}\left[2i\right]$) and ($X_{8}\left[2i-1\right]$, $X_{8}\left[2i\right]$) is the *i*-th group. The corresponding mark bit of the *i*-th group is LUT[i]. The value LUT[i] is set to be “1”, if and only if, in each block of the *i*-th group, the two indication bits are the same. Otherwise, LUT[i] is set to be “0”.

Note that the proposed approach focuses on reducing the data volume for the indications. With the advance of quantization techniques [31,32], low-bit weights and activations have been widely used in modern CNN accelerators. As a result, for modern CNN accelerators, the percentage of indications in the overall data volume is relatively enlarged. Thus, it is important to reduce the data volume for the indications.

**Figure 12:**
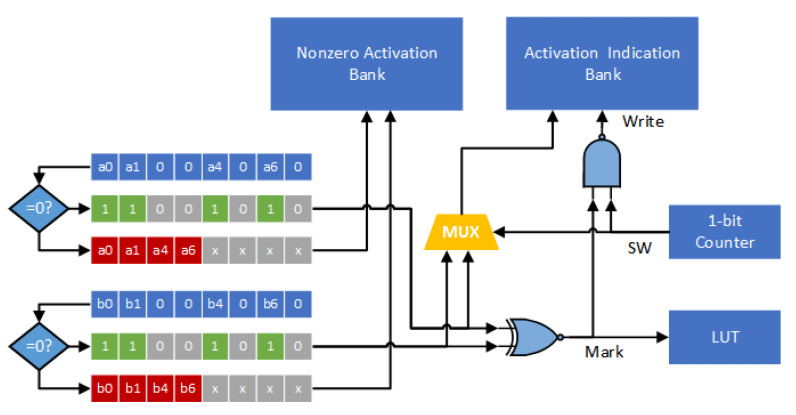
The proposed encoder circuit.

Next, we design an encoder, a decoder and an indexing module to support the proposed compression format. Figure 12 gives the corresponding encoder circuit. Here we consider eight channels at the same time. Thus, the bit-width of activation indication is 8. For each channel, we consider two activations (i.e., a block) simultaneously. Two zero-comparators are used to determine if the activations are zero or not. As shown in Figure 12, each zero-comparator scans eight activations, which are in the same group and from eight channels, and then records an indication string and nonzero activation values. All nonzero activation values are sent to the nonzero activation bank. The two indication strings, which are from two zero-comparators, are compared using an XNOR function. Note that the output of the XNOR function, called signal Mark, corresponds to the binary value of the mark bit (of this block). The binary value of the mark bit (i.e., the signal Mark) is stored in the LUT.

In Figure 12, the 1-bit counter (i.e., the signal SW) is used to control the mutiplexer (i.e., MUX). The signal Write determines whether the activation indication (i.e., the output of the multiplexer) is stored in the activation indication bank or not. Note that the binary value of the signal Write becomes “0” if and only if both signal SW and signal Mark are “1”. Therefore, if the binary value of the signal Mark is “0” (i.e., the two indication strings are different), the two indication strings are sequentially stored into the activation indication bank. On the other hand, if the binary value of the signal Mark is “1” (i.e., both the two indication strings are exactly the same), only one indication string is stored into the activation indication bank.

**Figure 13:**
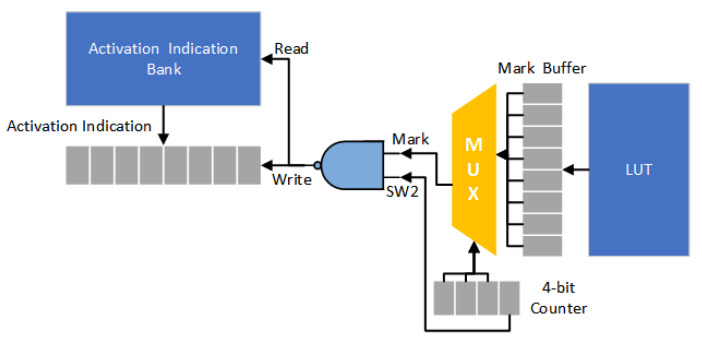
The proposed decoder circuit.

Figure 13 gives the corresponding decoder circuit. At each time, eight mark bits (i.e., one byte) are loaded from the LUT and then stored in the Mark buffer. The multiplexer is used to select a mark bit (as the signal Mark) from the Mark buffer. For each mark bit, we use two cycles to handle the loading of activation indications. Thus, a 4-bit counter is used to control the multiplexer. The signal SW2 corresponds to the least significant bit of the 4-bit counter. Thus, if the binary value of the signal Mark is “0”, two indication strings are sequentially loaded from the activation indication bank. On the other hand, if the binary value of the signal Mark is “1”, only one indication string is loaded from the activation indication bank.

**Figure 14:**
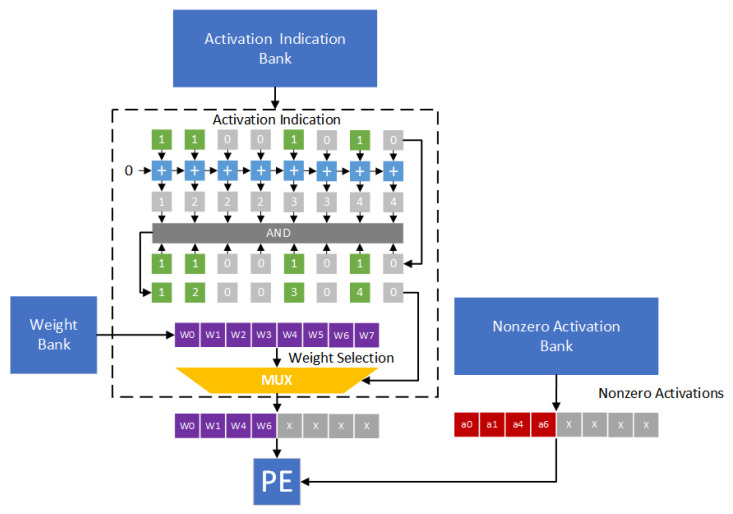
The proposed indexing module.

In the proposed approach, only nonzero activations are stored in the nonzero activation bank. Thus, there is a need to handle the alignment of irregularly distributed nonzero activations. Figure 14 gives the hardware design of the corresponding indexing module. In fact, the proposed indexing module is similar to the direct indexing module of Cambricon-X [9]. The main difference between the proposed indexing module and Cambricon-X is below: The proposed indexing module is to determine the indexes of nonzero activations, while Cambricon-X is to determine the indexes of nonzero weights.

We use Figure 14 as an example to explain the function of the proposed indexing module. In Figure 14, activations a0, a1, a4 and a6 are nonzero values. In other words, the indication string is 11001010. Each bit in the indication string is added to obtain an accumulated string. By enforcing the “AND” operation between the indication string and the accumulated string, the indexes of nonzero activations can be derived. Thus, as shown in Figure 14, weights w0, w1, w4 and w6 are selected. The pairs (a0,w0), (a1,w1), (a4,w4) and (a6,w6) are sent to the PE to perform convolution operations.

## 4. Experiment Results

We have used the TSMC 40 nm cell library to implement the corresponding hardware circuits, including the encoder, the decoder and the indexing module, to support the compression format. For comparisons, we also implemented the corresponding hardware circuits of previous works, including Cnvlutin [23], Cambricon-X [9] and Dual Indexing [26], to support their compression formats.

In the experiments, we assume the clock frequency is 1 GHz. In addition, we assume that the CNN accelerator is in the SIMD architecture [9,10,11,12,13,14,16,17,18,23,26,27]. In Cnvlutin [23], Cambricon-X [9] and Dual Indexing [26], the number of PEs is 16. Therefore, without loss of generality, here we also assume that the number of PEs is 16. Note that each PE requires an indexing module and a decoder. Thus, to support the proposed compression format, the circuit area overhead of a CNN accelerator is 16 indexing modules, 16 decoders and 1 encoder. Table 2 tabulates the circuit area overheads of different approaches (to support their compression formats in 16 PEs). Since Cambricon-X [9] focuses on the sparsity of weights, it does not need an encoder. Thus, Cambricon-X has the smallest circuit area overhead. On the other hand, compared with Cnvlutin [23] and Dual Indexing [26], the circuit area overhead of the proposed approach is smaller. The reason is that the indexing module of the proposed approach is simpler than those of Cnvlutin and Dual Indexing.

Then, we use six pre-trained CNN models in Keras [22], including vgg16, ResNet50, ResNet50v2, MobileNet, MobileNetv2 and DenseNet121, to test the effectiveness of the proposed approach. Note that we use the following methods to measure the memory traffic (i.e., the data movement to off-chip memory) and the power consumption. We extract the intermediate data during the CNN inference process (i.e., during the TensorFlow simulation). For each layer of a CNN model, the number of input channels, the input activations, the kernel weights, the output activations and the number of output channels are reported. According to this information, we can calculate the number of read accesses and the number of write accesses. As a result, the amount of memory traffic is derived. Note that the power consumption per read access and the power consumption per write access can be obtained from the TSMC 40 nm cell library. Therefore, we can also derive the total power consumption of all the memory accesses. Moreover, according to the number of input channels, the input activations and the kernel weights, we can derive the test patterns for the gate-level simulation. Based on gate-level switching activities (obtained by gate-level simulation), we can use Synopsys Design Compiler to report the power consumption of hardware circuit.

**Figure 15:**
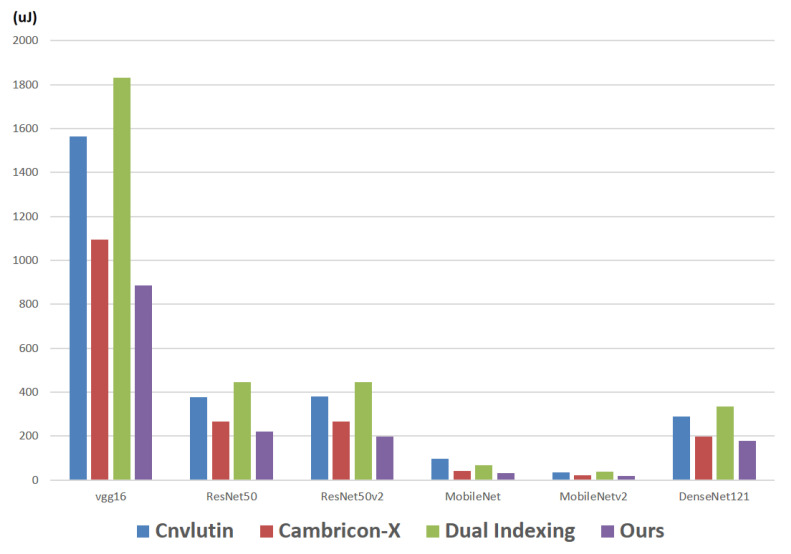
Energy consumption of the required hardware circuits in different approaches.

First, we report the energy consumption of the required hardware circuits (i.e., to support the compression format). Note that, in the proposed approach, the required hardware circuits are 16 indexing modules, 16 decoders and 1 encoder. Figure 15 makes comparisons on the energy consumption (of the required hardware circuits) among different approaches with respect to different CNN models. For example, in the CNN model vgg16, the energy consumption of Cnvlutin [23], Cambricon-X [9], Dual Indexing [26] and the proposed approach are 1563.1 μJ, 1095.0 μJ, 1832.5 μJ and 886.4 μJ, respectively.

As shown in Figure 15, in each CNN model, the proposed approach achieves the smallest energy consumption. The reason is because of the sharing of indication strings, in the proposed approach, the number of calculations in each indexing module can be greatly reduced. As a result, the power consumption (the energy consumption) can be greatly saved. With a detailed analysis to these six CNN models, we find that: compared with Cambricon-X [9], on average, the proposed approach can save 18% of energy consumption.

**Figure 16:**
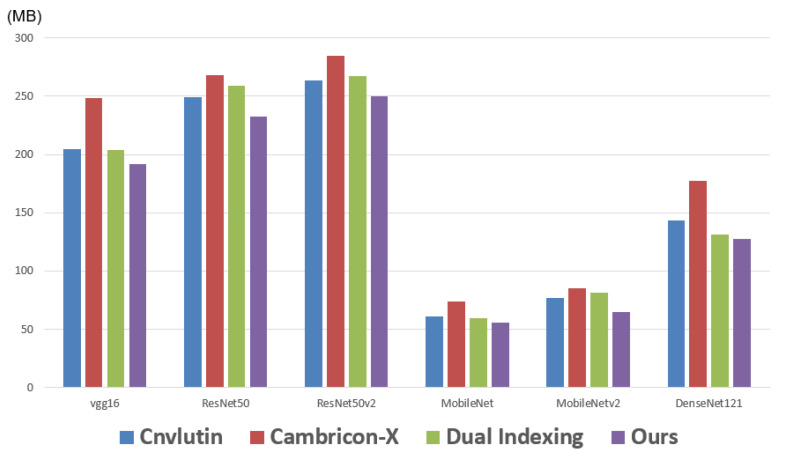
Memory traffic of different approaches.

Next, we report the memory traffic (i.e., the data movement to off-chip memory). Note that here we consider both the access of activations and the access of weights. Figure 16 makes comparisons on the memory traffics among different approaches with respect to different CNN models. As shown in Figure 16, in each CNN model, the proposed approach achieves the smallest memory traffic. The reason is that the proposed approach exploits both the sparsity and the similarity of activation values. Therefore, even compared with Dual Indexing [26], the memory traffic of the proposed approach is still smaller. With a detailed analysis of these six CNN models, we find that: compared with Dual Indexing, on average, the proposed approach can save 9% of memory traffic.

**Figure 17:**
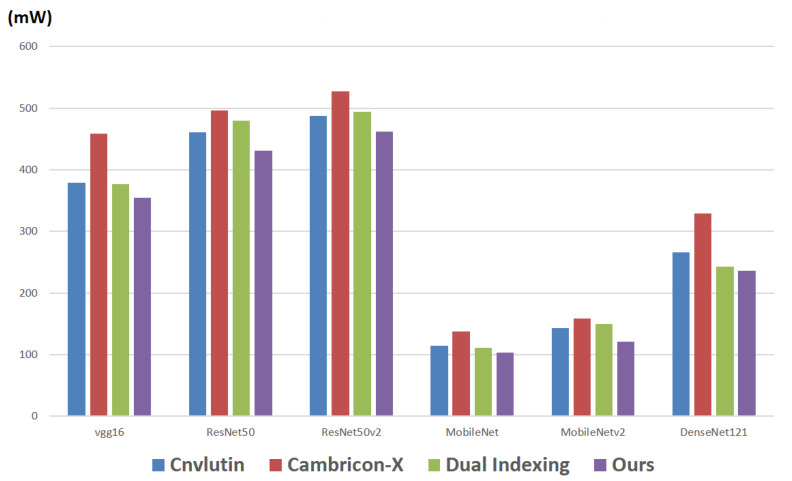
Power consumption of all the memory accesses in different approaches.

Finally, we report the power consumption of all the memory accesses. Note that here we consider both the access of activations and the access of weights. Figure 17 makes comparisons on the power consumptions of all the memory accesses among different approaches with respect to different CNN models. As shown in Figure 17, in each CNN model, the proposed approach also achieves the smallest power consumption.

We also implement a C program, which is integrated into the TensorFlow simulation, to simulate the behaviors of the different approaches (i.e., different compression mechanisms) during the CNN inference process. Table 3 tabulates the top-1 accuracies with respect to different approaches. Note that these approaches do not introduce any accuracy loss. Therefore, as shown in Table 3, these approaches achieve the same top-1 accuracies in each CNN model.

## 5. Conclusions

This paper demonstrates both the sparsity and the similarity of feature maps. Based on these observations, we propose a block-based compression format, which utilizes both the sparsity and the similarity, to reduce the data volume of indications. Compared with the dual indexing mechanism, the experiment results show that the proposed approach can save 9% of memory traffic.

Moreover, we also develop the corresponding hardware circuits, including an encoder, a decoder and an indexing module to support the proposed compression format. Compared with the corresponding hardware circuits of previous works (to support their compression formats), the power consumption of the proposed approach (i.e., the corresponding hardware circuits of the proposed compression format) is the smallest.

As low-bit activations are used in modern CNN accelerators, the percentage of indications in the overall data volume is relatively enlarged. Thus, it becomes more important to reduce the data volume for the indications. With the trend of low-bit quantization in the edge computing, the proposed approach (for reducing the data volume of indications) is promising.

## Figures and Tables

**Table 1:** An analysis to the activation sparsity of CNN models.

vgg16	ResNet50	ResNet50v2	MobileNet	MobileNetv2	DenseNet121
49.0%	33.1%	39.5%	49.4%	65.7%	48.7%

**Table 2:** The circuit area overheads of different approaches (in 16 PEs).

Cnvlutin [23]	Cambricon-X [9]	Dual Indexing [26]	Ours
16,208 $\mu m^{2}$	12,112 $\mu m^{2}$	30,108 $\mu m^{2}$	13,599 $\mu m^{2}$

**Table 3:** Top-1 accuracies of different approaches.

CNN Model	Approach			
	Cnvlutin	Cambricon-X	Dual Indexing	Ours
vgg16	71.3%	71.3%	71.3%	71.3%
ResNet50	74.9%	74.9%	74.9%	74.9%
ResNet50v2	76.0%	76.0%	76.0%	76.0%
MobileNet	70.4%	70.4%	70.4%	70.4%
MobileNetv2	71.3%	71.3%	71.3%	71.3%
DenseNet121	75.0%	75.0%	75.0%	75.0%

## Data Availability

The data used to support the findings of this study are included in this paper.
